# Adaptive Wireless Network Management with Multi-Agent Reinforcement Learning

**DOI:** 10.3390/s22031019

**Published:** 2022-01-28

**Authors:** Ameer Ivoghlian, Zoran Salcic, Kevin I-Kai Wang

**Affiliations:** Department of Electrical, Computer and Software Engineering, The University of Auckland, Auckland 1010, New Zealand; z.salcic@auckland.ac.nz

**Keywords:** large scale networks, congestion, reinforcement learning, multi-agent, LoRaWAN, fairness, application awareness

## Abstract

Wireless networks are trending towards large scale systems, containing thousands of nodes, with multiple co-existing applications. Congestion is an inevitable consequence of this scale and complexity, which leads to inefficient use of the network capacity. This paper proposes an autonomous and adaptive wireless network management framework, utilising multi-agent deep reinforcement learning, to achieve efficient use of the network. Its novel reward function incorporates application awareness and fairness to address both node and network level objectives. Our experimental results demonstrate the proposed approach’s ability to be optimised for application-specific requirements, while optimising the fairness of the network. The results reveal significant performance benefits in terms of adaptive data rate and an increase in responsiveness compared to a single-agent approach. Some significant qualitative benefits of the multi-agent approach—network size independence, node-led priorities, variable iteration length, and reduced search space—are also presented and discussed.

## 1. Introduction

Wireless networks are ubiquitous, more so every day. The projected growth of the Internet of Things (IoT) will enable a range of applications and scenarios, such as smart grids, environmental monitoring, intelligent transport, pervasive healthcare, and agricultural monitoring. These applications demand thousands of devices, but with this increased scale, the frequency of congestion also increases. Congestion is a key issue for all networks operating in license-free radio bands, but low-power wide-area networks (LPWANs) [1] are particularly susceptible. LPWANs are characterised by low data rate (tens of kbit/s), low power (multi-year battery life), long range (city wide), and high node count (thousands of nodes). LoRaWAN is one such protocol.

The LoRa radio protocol, on which the LoRaWAN protocol sits, utilises a star topology. It targets uplink-focussed applications, such as data collection from geographically distributed sensor nodes. LPWANs such as LoRaWAN typically have limited network bandwidth [2], and lack network-wide coordination, which is acceptable at small scales, but becomes a serious limitation when scaled up. Low data rates mean that packets spend a long time on air. This can be in the order of seconds depending on configuration. When the time on air (ToA) is this long, the messaging rate must be very low for a network containing thousands of nodes, or else there is a significant probability of packet collision.

In addition, this is not a static problem, and it will get worse with the increases in network size and traffic. Factors, both internal and external to the network, create a dynamic and unpredictable radio environment. Internally, network growth, node mobility, and traffic variability due to changing application requirements undermine the effectiveness of statically configured network parameters. Externally, changes in the propagation environment and interference from co-located networks are both inevitable and uncontrollable. This variability in network conditions, which can be termed “network dynamics”, is a problem for all large networks. The unpredictability of network dynamics makes them difficult to overcome in a statically configured network. The low data rate and low-power characteristics of LPWANs preclude any significant top-down network management. Nodes typically determine when and with which settings they transmit, and downstream control messaging is severely limited by duty-cycle regulations. This makes LPWANs particularly susceptible to network dynamics.

To ensure good utilisation of limited wireless resources, and reduce network congestion for large scale networks, dynamic and automated network management is necessary. However, control and optimisation of dynamic, large scale networks is reaching unprecedented levels of complexity [3]. Large scale networks are simply too large to rely on manual control and static configuration.

Existing network management techniques, especially in the context of LoRaWAN, emphasise the optimisation of a single metric. This is typically packet delivery ratio (PDR) or packet error rate (PER). However, real-world applications often require a balance of multiple, conflicting performance metrics. An example of this is the conflict between PDR and energy consumption. Additionally, large scale networks are not limited to a single application, and it is reasonable to expect that there may be a variety of performance goals within a network. An abstract performance metric is required that captures the unique requirements of an application, consisting of multiple physical metrics. Therefore, in addition to the ability to automatically adapt to network conditions, it is also important that the process of managing the network be “application aware”.

LoRaWANs are vulnerable to the issue of congestion caused by scale and network dynamics. This congestion logically results in an uneven allocation of resources [4]. This unfairness leads to inefficient use of the available network capacity. Illustrating this point, a node which cannot access adequate network capacity and is affected by congestion might respond by lowering its data rate or increasing its transmission power (TXP); this only serves to further congest the network, degrading the performance of other nodes. It is vital that, as much as possible, fairness is considered.

In summary, any solution to congestion in large scale wireless networks must be:

1. Autonomous—large scale networks are too complex to rely on manual control and static configuration.
2. Adaptive—the behaviour of co-located networks, node mobility, network growth, traffic variability, or the propagation conditions (network dynamics) are uncontrolled and unpredictable.
3. Application aware—real-world applications often require a balance of multiple conflicting performance metrics, and large networks often host multiple applications, with different requirements.
4. Fair—uneven resource allocation is a logical consequence of congestion, resulting in nodes fighting for resources, which is an inefficient use of the available network capacity.

This matter can be framed as an optimisation problem. However, without an exact mathematical model, the problem space, which is huge, must be efficiently explored to find an optimal solution. Machine learning approaches are well suited to “model-free” problems such as this. Reinforcement learning (RL) is a machine learning paradigm which is a good match for control tasks. It performs well when the problem space is large, and when there is a temporal element to the search, such as delayed reward [5]. In the context of this problem, there is a natural delay between changing radio parameters on a node and the resultant change in performance. This phenomenon becomes more acute as the network scale increases, where the performance of a node is impacted by the behaviour of many other nodes. Searching the problem space for an optimal solution is an iterative process, taking time to settle on an acceptable parameter configuration. RL’s accumulated reward element is appropriate for the online nature of this real world scenario. A range of RL algorithms have been proposed and developed, proving a variety of policies for exploring the problem space. These policies are suited to real world applications where exhaustive searching is too time consuming. Algorithms which employ function approximation are even more appropriate when the problem spaces are intractably large or complex, providing useful results with only partial exploration of the problem space. The RL algorithm utilised by this study is the deep Q-network (DQN) algorithm [6].

In this paper, we present an autonomous and adaptive wireless network management framework. The proposed framework utilises multiple, distributed, deep RL agents, to achieve efficient use of network resources. A novel reward function incentivises the satisfaction of node-specific application requirements, while also satisfying the global objective of fairness. Responsiveness is improved compared to a related single-agent approach, and network size independence, node-led priorities, and variable iteration lengths are made possible. Together, these contributions address the issue of congestion in large scale wireless networks, while meeting the requirements of autonomy, adaptivity, application awareness, and fairness. Experimental results demonstrate these capabilities, and compare the performance of the proposed approach against those of existing approaches.

Section 2 provides an overview of related works; Section 3 provides the background of the case study; Section 4 describes the proposed approach; Section 5 presents the experimental set-up, results of the evaluation, and discussion; Section 6 sums up the contributions and directions for future work.

## 2. Related Work

The literature review is presented in two sections. The first part examines non-RL-based approaches to managing and optimising the performance of LoRaWANs. The second part examines RL-based approaches to managing wireless networks in general.

### 2.1. LoRaWAN

The assignment of spreading factors (SFs) in a network depends on the positions of the respective nodes relative to the base station (LoRaWAN gateway), and the state of the radio communication environment. LoRa proposes a mechanism which LoRaWAN adopts for facilitating the automated assigning of SF. This is called adaptive data rate (ADR) [7]. ADR effectively maximises PDR in certain scenarios. However, Li et al. [8] and Slabicki et al. [9] demonstrate that ADR is best suited to static and stable link environments, and struggles when network dynamics are in play. It also responds poorly to congestion, where there are high levels of packet collision, as shown by Kim et al. [10].

Sallum et al. [11] set out to improve the performance of LoRaWANs by framing it as an optimisation problem, and using mixed integer linear programming (MILP) to find an optimal SF and carrier frequency (CF) set. Their MILP problem formulation, which uses an approximation algorithm, minimises the utilisation of CF and SF combinations, thereby minimising the chance of collisions. This assumes SF orthogonality. Compared with standard LoRaWAN, data extraction rate (DER) and energy consumption are improved.

Dix-Matthews et al. [12] explored the trade-off between reliability and energy consumption, specifically data delivery ratio versus energy consumption per packet. Their primary focus is on maximising PDR, and reducing energy consumption is a secondary objective. They proposed and demonstrated that the selection of LoRa radio parameters which reduce energy consumption, combined with payload replication and forward-error-correction, can improve data extraction while maintaining comparable energy consumption.

Cuomo et al. [13] proposed two approaches for allocating SFs to nodes in a network. The first, EXPLoRa-SF, distributes SFs across nodes uniformly. This takes advantage of LoRa’s SF orthogonality, which ADR does not. Their second approach, EXPLoRa-AP, considers that lower SFs consume less time on air (ToA), and can therefore accommodate proportionally more nodes for the same level of network utilisation. They assert that distributing nodes evenly without considering this would load SFs unevenly. Their simulation results demonstrate an improvement in DER.

Abdelfadeel et al. [14] derived a data rate distribution with the goal of achieving fair probability of collision between nodes in a LoRaWAN. They then proposed a TXP control algorithm, FADR, based on this distribution. FADR adjusts TXP to achieve uniform received signal strength across all nodes, thereby reducing the occurrence of the capture effect. This makes the choice of SF independent of the node’s distance from the base station. Using the LoRaSim [15] simulator, they demonstrated that FADR achieves an almost uniform DER from all nodes in the network, independent of distance from the base station.

### 2.2. Reinforcement Learning

He et al. [16] applied deep RL to the task of optimising a cache-enabled opportunistic interference alignment network. Their simulated results demonstrate agent convergence and improvements in the network’s average sum rate (total system packet throughput) and energy consumption compared with existing schemes.

Yu et al. [17] proposed a MAC protocol, named DLMA, for heterogeneous environments containing multiple networks. DLMA uses deep RL to maximise the sum-rate of all coexisting networks. They then replaced this objective with another, named α-fairness. Their evaluation demonstrates that DLMA achieves near-optimal performance without knowledge of the MAC protocols of the coexisting networks. This was carried out in saturated scenarios in which all nodes always have packets to transmit.

Zhu et al. [18] asserted that poor transmission efficiency in crowded spectrum is a barrier to the implementation of IoT applications. They proposed applying deep RL to the task of selecting transmission channels, transmission modes, and buffer. Their simulation demonstrated that their approach achieves similar performance to the strategy iteration algorithm. The strategy iteration algorithm yielded the optimal solution, but is impractically complex for wireless networks.

Ilahi et al. [19] proposed using deep RL (specifically double DQN) to configure the SF and TXP of nodes at runtime. As in this study, the action space consisted of two adjustable parameters, SF and TXP. Their state space utilisesd those same adjustable parameters, together with the node distance from the gateway. The reward function considered PDR, airtime, and min-max normalised TXP, using constants to assign weights to these three components. They evaluated their proposed approach with a network containing 100 nodes. A significant improvement in PDR performance was demonstrated compared with the approach implemented in the LoRa-MAB simulation framework [20] when node mobility was introduced.

Nisioti et al. [21] proposed a MAC design for wireless sensor networks utilising the irregular repetition slotted ALOHA protocol. Like ours, their approach uses multiple RL agents, deployed on the nodes, in a decentralised manner. However, it utilises a tabular RL algorithm (Q-learning), limiting the size of the state–action space and constraining the observability to information available locally to the node. They accelerated learning using virtual experience. Analysis demonstrates that their system finds a near-optimal solution for the IRSA protocol.

Similarly to our study, Yu et al. [22] proposed applying multi-agent RL to the task of managing a LoRa based network. However, their explicit goals were to improve reliability and reduce power consumption; the conflict between these two metrics was not addressed directly. As in this study, the action space consisted of two adjustable parameters, SF and TXP. However, the state space was much simpler, composed of a binary indicator of connection reliability for each node. This kept the search space small, allowing the use of tabular RL algorithms, in this case Q-learning. The reward function attempts to balance connection reliability with normalised TXP, using constants to assign weights to the two halves of the function. They evaluated the proposed approach with a small network containing five agents or nodes. This was compared to a network which is statically configured according to distance from the base station. The results demonstrated improvements in both reliability and power consumption.

Fedullo et al. [23] used the SARSA algorithm for LoRaWAN management. SARSA is a table-based method, and so it is constrained to relatively small problem spaces. The authors addressed this by “decoupling the training activity from the specifically considered node deployment”, and sharing a Q-table between all nodes. This was likely made possible due to the inclusion of a signal-to-noise ratio term in the state definition and reward function, generalising the learning for node position. Like other related work, they utilised SF and TXP as the action space. The approach was evaluated against ADR, and demonstrated improvements in both mean and minimum DER. They proposed adding a back-off mechanism to the action space in future work.

A common characteristic of the related work is the limited scope of their reward functions. In single-agent approaches, they only considered network performance, and in multi-agent approaches they focused on the performance of the key node. The synergy of these two perspectives was not considered. Application awareness and fairness are not directly addressed, limiting their applicability to some application scenarios, and their ability to balance the needs of all nodes in a network.

In our previous work [24], we proposed applying single-agent RL to the task of automatically managing a large scale LoRaWAN. Application awareness is a goal which is reflected in the devised reward function. Experimental results revealed that the RL system copes much better than ADR when interference is present. Its application awareness was also displayed with the use of different application requirements. In short, it demonstrates that deep RL can be applied to the task of large scale, automated, wireless network management. However, the single-agent approach suffers from the limitation of the global performance perspective. The simple reward function also restricts the effective integration of the fairness goal.

This study addresses the shortcomings of the existing approaches. A multi-agent approach is taken, and a novel reward function, combined with a suitably defined state space, enables the synergistic combination of both node and network performance objectives, including application awareness and fairness.

## 3. LoRaWAN

The LoRaWAN protocol was targeted in this research because of its susceptibility to the issues of network dynamics and scale. To put it into the context of the proposed solution, two aspects of the network must be defined: the adjustable parameters which can be controlled to optimise performance, and the metrics with which performance can be measured.

### 3.1. Adjustable Parameters

While there is no limitation on the number of parameters that can be adjusted, increasing the number of parameters makes the problem non-linearly more complex. For this study, two parameters were chosen, namely, SF and TXP.

#### 3.1.1. Spreading Factor

LoRa’s quasi-orthogonal [25,26,27] SF feature enables the decoding of multiple concurrent packets, so long as they are using different SFs and have similar power levels at reception. The LoRaWAN regional parameters dictate which SFs, bandwidths, and frequencies are available. This research adopted SF7 through SF12. Each increment in SF provides an additional 2.5 dB of link budget, in the form of coding gain. This comes at the cost of reduced data rate, and hence increased ToA. SF12 provides 12.5 dB of gain compared to SF7, which corresponds to an approximately four times increase in transmission range in free space, or an increased likelihood of successful packet decoding at the same range. However, it also increases ToA by a factor of 18, hence increasing the probability of packet collision. The adjustment of this parameter controls the trade-off between ToA and link budget, but more importantly, it affects the balance between individual node performance and whole network performance.

#### 3.1.2. Transmission Power

Adjusting TXP directly controls the link budget, which itself has a direct impact on successful packet decoding. For a packet to be successfully decoded, it must arrive at the receiver with a signal level greater than the receiver’s effective sensitivity. This research utilises the Semtech SX1276 transceiver [28], which supports TXP values between –4 and 14 dBm, in 2 dB steps. Moving from the lowest value to the highest yields 18 dB of additional link budget, increasing the likelihood of successful packet decoding, or an approximately eight times increase in range in free space. Even in the case of packet collision, packets sent with higher TXP levels have a greater chance of dominating others due to the capture effect. The unnecessary increase in TXP at the node level increases energy consumption. However, at the network level, it can prevent other nodes from successfully delivering packets, using their current transmission parameters. This gives rise to competitive behaviour between nodes, and will result in inefficient use of network capacity.

### 3.2. Performance Metrics

Real-world applications often require a balance of multiple conflicting performance metrics. However, the performances of most wireless networks are measured using just one metric, most commonly PDR. For this study, in keeping with the goal of application awareness, two conflicting performance metricwere selected: PDR and energy consumption per packet (ECP). Placing emphasis on one or the other allows a diverse range of applications to be catered for.

#### 3.2.1. Packet Delivery Ratio

PDR is a key measure of node performance. It is influenced by a range of factors, from radio transmission and reception, to network layer effects. It captures the impacts of these factors and provides an indication as to the overall communication performance. Specifically, the PDR is defined in (1), as the ratio of successfully delivered packets to total packets sent.

(1) $$\mathrm{PDR}=\frac{packets\;delivered}{packets\;sent}.$$

PDR is computed based on the percentage of packets which arrive correctly at the base station and can easily be measured using packet counters.

#### 3.2.2. Energy Consumption per Packet

The second metric, ECP, is particularly important in applications with battery powered nodes. Battery replacement in large scale networks is a significant burden and must be avoided by minimising the amount of energy consumed during node operation. There are a variety of ways to measure energy consumption. In this study, the application agnostic ECP metric was utilised. It can be measured on the node using (2).

(2) $$\mathrm{ECP}=V_{supply}\times I_{supply}\times T_{packet},$$

where $V_{supply}$ is the supply voltage, typically a constant value from a regulated battery source; $I_{supply}$ is the current draw based on $V_{supply}$ and the configured TXP; and $T_{packet}$ is the transmission time of a packet.

## 4. Multi-Agent Reinforcement Learning

### 4.1. Architecture

**Figure 1:**
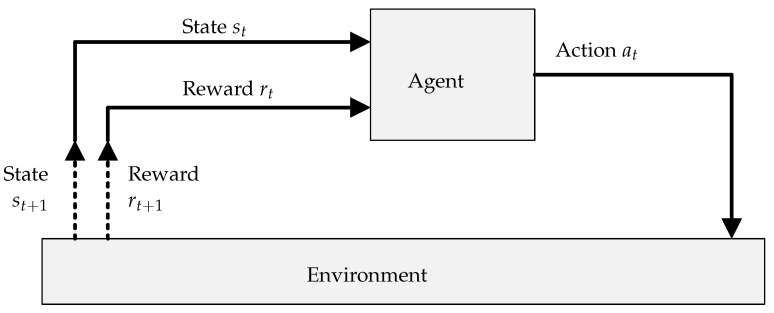
Simplified RL agent diagram.

At the most basic level, an RL system consists of an agent interacting with an environment. This relationship is illustrated in Figure 1. The agent’s goal is to simultaneously determine and follow a policy which maximises its cumulative return over time. In many contexts, this can alternatively be described as mapping the current state of the environment to the best action to take in that state.

A simplified description of the RL learning process is as follows:

1. Given the current state $s_{t}$, the agent selects an action $a_{t}$ according to its current policy.
2. The environment transitions to the next state $s_{t+1}$ in response to action $a_{t}$ and emits a reward signal $r_{t+1}$.
3. The agent observes the reward $r_{t+1}$, updates its policy, and returns to step 1.

A timely change in the environment, a complete state space, and a suitable reward function expedite the policy update or learning process.

**Figure 2:**
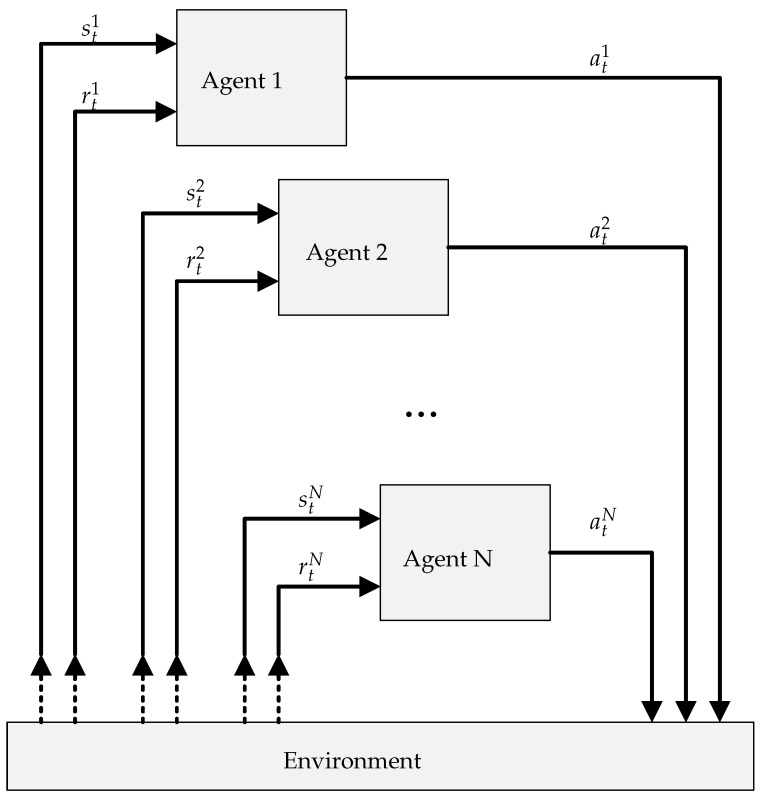
Simplified RL multi-agent diagram.

Compared with single-agent systems, multi-agent systems differ in that there are multiple agents interacting with a common environment, as illustrated in Figure 2. In multi-agent systems, the agents can all share the same perspective of the environment, where they all receive the same state information, or they can each have a distinct, partial view of the environment. This study considers the latter, due to the limited information each node has about the environment. The agent actions are unique, though they all affect the same environment, either through some external merging function or by affecting different parts of the environment. This study again considers the latter, which is aligned with the distributed nature of the approach.

In multi-agent systems, depending on the scenario, it can be difficult to directly associate a reward with the actions of a specific agent. This is because the change in state is the consequence of the combined actions of all agents. This makes the mapping of states to actions, described above, challenging. Despite this, there are advantages to the multi-agent approach which make it beneficial in some scenarios. In the context of the network management scenario, the benefits include:

- Independence of network size: In a single-agent approach, the agent environment must be pre-defined according to the number of nodes in the network. With multiple agents, the network can be an arbitrary size, even growing and shrinking during operation, without redefining and retraining agents.
- Node-led priorities: Multiple, competing goals, which are difficult to capture with a single reward function, can be divided up among multiple agents. Nodes can define and implement their own application requirements. This is particularly useful for multi-tenanted networks.
- Variable iteration length: Nodes with more computational power, or more energy capacity, can utilise shorter learning iterations, improving responsiveness, compared with energy or computationally constrained devices.
- Reduced search space: With multiple agents, the search space is significantly smaller per agent, reducing the complexity and computational requirements, and enabling implementation on resource constrained hardware.

**Figure 3:**
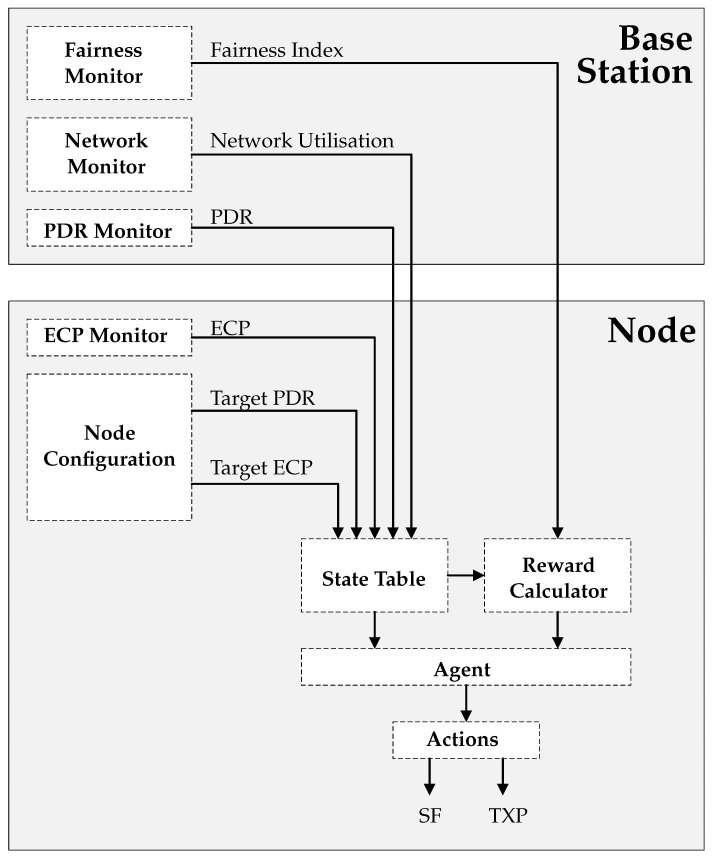
System model and relationship between agent, base station, and node.

The benefits above make the case for applying multi-agent RL to the task of large scale wireless network management. The solution requires a range of components, hosted on both the node and the base station, which support the operation of the agent. Figure 3 illustrates the various components of the system, with arrows indicating the flow of data between these components. The following subsections describe the functionality of each of these components in greater detail.

### 4.2. Base Station Components

#### 4.2.1. PDR Monitor

Due to the uplink focussed nature of the network, the node has limited visibility regarding whether packets have been successfully delivered or not. PDR is most easily measured at the base station, which is receiving the packets. The PDR monitor component is therefore hosted on the base station. It keeps track of packet counters and uses this to calculate the PDR for each node according to (1). Algorithm 1 describes this process. It fills a buffer *K* with 1 for successfully received packets, or 0 for missed packets (calculated with packet counters), using a circularly incremented index *i*. It returns a PDR value which is the fractional mean of the buffer values. The calculated PDR value is sent to the relevant node after every 20 uplink packets. This parameter is adjustable, but 20 was selected to maintain some consistency with ADR, to which this approach will be compared. Any change in transmission parameter (SF, TXP, etc.) on the node resets the process, and another 20 packets must be received before PDR is sent.
**Algorithm 1:** Base station: PDR calculation.**  Data:** PDR monitoring buffer: *K*, buffer index: *i*, buffer length: *L*, current packet counter value: *C*, previous packet counter value: *p*

**  Result:**
*PDR* value 

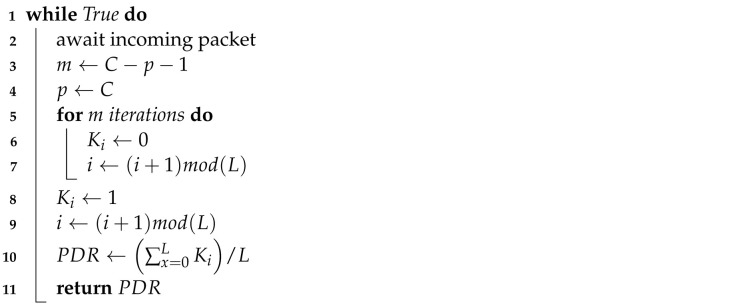


#### 4.2.2. Network Monitor

A complicating factor when moving from a single-agent approach to a multi-agent approach with partial observability of the environment is that environmental variables which impact the reward function may be excluded. This disrupts the agents’ ability to map states to actions. In a network, the performance, and hence reward, of a node is directly affected by the network state. This includes the behaviour of other nodes in the network, other co-located networks, or the propagation environment.

All these factors fundamentally impact the base station’s ability to decode a packet. The packet under any of these influences can be described as lacking sufficient signal-to-noise ratio throughout its time on air. The effects of these influences can therefore be represented as “noise”. The network monitor component quantifies this noise and provides it to the agent, helping to fill the gaps in its observation of the environment. We refer to this metric as “network utilisation”, as it gives an indication of how loaded the communication channel is.

The measurement can be made directly at the base station, in the form of received signal strength indicator (RSSI). RSSI can be measured by the base station’s radio. It provides a measure of the incoming signal power at the RF input port (within the receiver bandwidth). The RSSI value is smoothed using a moving window filter, resulting in the network utilisation metric. Algorithm 2 describes this process. For this implementation, it is executed every 30 s, and the buffer length is 60, resulting in window of 30 min. Network utilisation is continually updated on the base station, and is sent to the nodes along with PDR.
**Algorithm 2:** Base station: network utilisation.**  Data:** RSSI reading: γ, sample buffer: *J*, buffer index: *i*, buffer length: *L*

**  Result:** Network utilisation: *U*

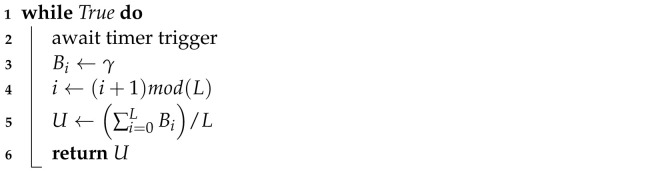


#### 4.2.3. Fairness Monitor

The main purpose of the fairness monitor is to calculate a measure of network fairness, on an ongoing basis, and to provide this value to the nodes to be used in their reward calculation. This measure of fairness, shown in (3), is a modified version of Jain’s Index [29], which has been altered to be application aware. This formulation differs from Jain’s Index in that the $x_{i}$ term, which represents throughput in a wireline TCP network, has been replaced with node performance $P_{n}$, which is defined later in (4), Section 4.3.4. Fairness is continually updated on the base station, and is sent to the nodes along with PDR.

(3) $$f=\frac{{\left(\sum P_{n}\right)}^{2}}{N\sum {(P_{n}}^{2})}.$$

Fairness *f*, represents how equally all nodes are meeting their application requirements, as captured by $P_{n}$. *N* represents the number of nodes in the network. Fairness values approaching 1 reflect a network with a high degree of fairness, and values approaching 0 reflect a network with low fairness. The impetus to unfairness is resource contention, which indicates insufficient network capacity.

### 4.3. Node Components

#### 4.3.1. ECP Monitor and Node Configuration

The node hosts two components, external to the RL agent, which feed its state table and support its operation.

Unlike PDR, ECP can be computed directly by a node itself. This is calculated using (2). ECP is fed directly into the state table, and is in-turn, used for reward calculation.

The node configuration component is a local store for the application-specific metric requirements. It is maintained as a separate module to allow the requirements to be changed on the fly if necessary. These are:

- Target PDR: The target PDR value of the application.
- Target ECP: The target ECP value of the application.

#### 4.3.2. State Table

An ideal RL state space must represent all possible states the environment can exist in. In the context of a wireless network, this includes every variable which might have an influence on the performance of the network. Defining the state space is not always straightforward. On one hand, the state table must capture as much relevant information about the environment as possible, because excluding an important variable disrupts the agent’s ability to map states to actions. However, practically, there needs to be some bounds on the size of the state space. Every new variable increases the dimension of the state space, which the agent is trying to explore.

For this study, the state table contains:

- PDR: The packet delivery ratio of the node, measured and supplied by the PDR monitor on the base station.
- ECP: The energy consumption per packet, measured by the ECP monitor on the node and inverse normalised.
- Target PDR: The target PDR value of the application.
- Target ECP: The target ECP value of the application.
- Network Utilisation: The network utilisation metric, calculated and supplied by the network monitor on the base station.

#### 4.3.3. Actions

As already discussed in Section 3.1, the most direct way to affect node and network performance is by adjusting the SF and TXP parameters of the nodes. These two parameters therefore comprise the action space of the agent.

#### 4.3.4. Reward Calculator

The reward function provides the reinforcement signal that encourages an agent to choose favourable actions given the current state. This must reflect the two goals: application awareness and fairness. As discussed, associating the actions of a node to changes in the environment can be difficult. This makes designing the reward function non-trivial. In the context of this problem, two approaches were initially considered:

- Global Reward: The reward function reflects the average performance of the entire network. The downside of this approach is that, in a large network with many agents, the actions of a single agent have only a minor effect on the global reward. Without a strong and timely reward for taking actions, an individual agent is not motivated to take good actions (lack of reinforcement). This reward function approach potentially creates lazy agents.
- Local Reward: The reward function reflects the performance of the respective node. The downside of this approach is that agents are only rewarded for improving the performance of their corresponding node. The performance of the network as a whole is not directly captured. This reward function approach potentially creates selfish agents.

To address the shortcomings of these approaches, a hybrid reward function can be utilised. The lazy agent lacks the reinforcement necessary for it to seek out an optimal solution. Starting with the selfish agent and adding a global objective to make it less selfish is a logical path forward.

Individual node performance $P_{n}$ is shown in (4). It is the product of the satisfaction *Q* for each metric *m*, with *M* representing the metric count. In this study, these metrics are PDR and ECP. $Q_{m}$ is the ratio of the current measured metric value $C_{m}$ to the configured target metric value $T_{m}$, capped at 1, as shown in (5). $P_{n}$ effectively captures how well the node is meeting its application requirements, satisfying the application awareness goal. This is the local component of the reward function.

(4) $$P_{n}=\prod_{m=0}^{M-1}Q_{m},$$

(5) $$Q_{m}=\left\{\begin{array}{cc} \frac{C_{m}}{T_{m}}, & C_{m}<T_{m}\\ 1, & C_{m}\geq T_{m} \end{array}\right..$$

The global component of the reward function is fairness *f*, as shown in (3). It prevents individual nodes from being selfish.

Where there is not enough network resource available for all nodes to meet their application requirements, some nodes inevitably perform more poorly than others. Low levels of fairness indicate insufficient network capacity. In these cases, it is desirable for nodes to reduce their network utilisation, which likely results in a reduction in $P_{n}$. Naturally, in the interest of improving fairness, nodes with higher $P_{n}$ should have a greater incentive to reduce network utilisation. When there is excess network capacity available, indicated with high levels of fairness, nodes should be encouraged to increase their $P_{n}$ without limit. It follows then, that when fairness is moderate, a moderate $P_{n}$ should be encouraged for all nodes, where nodes with high $P_{n}$ are encouraged to reduce network utilisation, and nodes with low $P_{n}$ are encouraged to increase network utilisation. Such an objective is synonymous with the goal of fairness.

**Figure 4:**
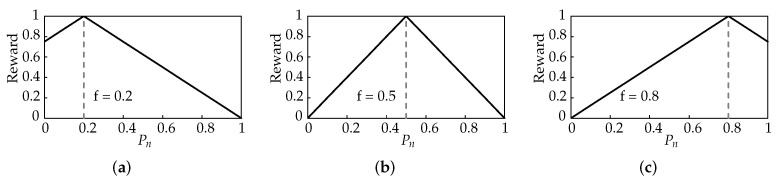
Comparison of triangular reward function for different fairness values. (**a**) f=0.2. (**b**) f=0.5. (**c**) f=0.8.

To achieve this, the reward function is designed to take a triangular form illustrated in the examples in Figure 4a–c. Note how the different target $P_{n}$ values ($P_{n}$ values where the reward is at its maximum) change according to network fairness.

To capture the varying effect of network fairness on individual reward functions, the reward function is formulated in the following general form in (6).

(6) $$R=\frac{1-2|P_{n}-f|+\alpha }{\beta }.$$

The $1-2|P_{n}-f|$ term produces the triangular function, with the peak at *f*. In order to keep the reward function between 0 and 1, an offset α and scaling β factor are included in the function. They too must take a similar form as shown in (7) and (8) to tailor to the varying effect of network fairness, *f*.

(7) α=|1−2f|,

(8) β=1+|1−2f|.

While $P_{n}$ is specific for each node, and agent actions are quickly reflected in the reward, *f* is equally affected by all nodes in the network, and in a large scale network, the actions of one agent are unlikely to have a significant effect on the reward. Instead, all agents in the network work together, without coordinating, to improve this metric. The two components of the reward function, network fairness and node performance, are combined to address the goals of application awareness and fairness.

### 4.4. Agent Operation

**Figure 5:**
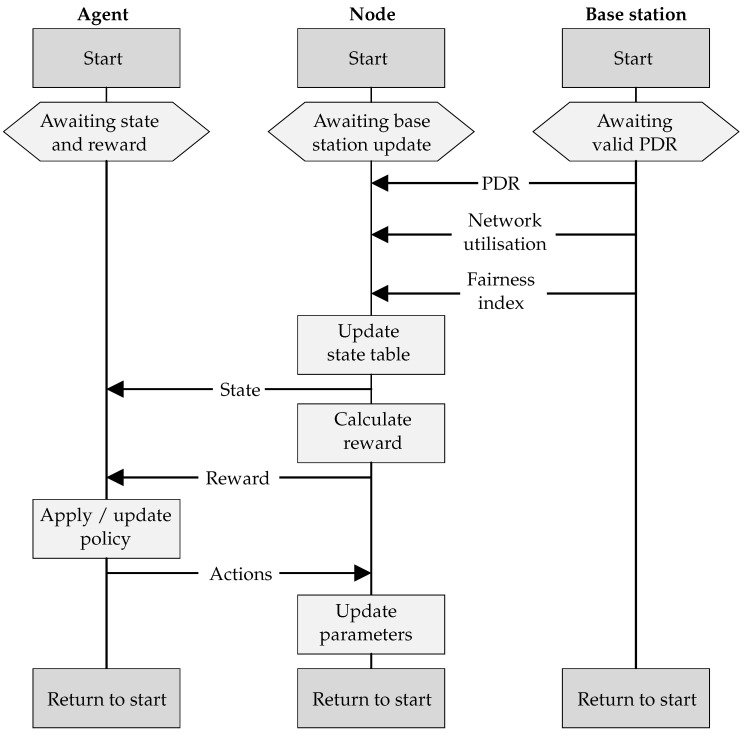
Message sequencing among the agent, node, and base station.

To illustrate the various parts supporting the agent learning process, the sequence diagram of communication among the base station, a node, and its corresponding agent is shown in Figure 5. Although the agent is hosted within the node, it is presented independently here to illustrate its logical separation.

The agent component awaits an updated state table and reward signal from the node, before issuing a set of actions, and then moving to the start of the next iteration. The node awaits PDR, network utilisation, and fairness index from the base station before updating the state table, calculating the reward, and sending both to the agent. It then receives updated actions from the agent and updates its parameters, before moving to the start of the next iteration. The base station sends PDR, network utilisation, and fairness information to the node. It does this once 20 consecutive packets have been received from the node, after which it moves to the start of the next iteration and awaits another 20 packets.

LoRaWANs are uplink focussed, with optional downlink windows following every uplink packet. The PDR, network utilisation, and fairness index are sent from the base station to the nodes in these windows. Nodes that do not implement an RL agent can still participate in the network, either by indicating that they do not require the PDR, network utilisation, and fairness data, or by ignoring it. Nodes can also alter the frequency with which they receive this data, by modifying the number of uplink packets per update, via a request to the base station. This controls the trade-off between responsiveness, computational power, and energy consumption.

Classical RL algorithms, such as Q-learning, rely on tables to record the expected return values for every state–action pair. However, when the state space contains more than just a couple of dimensions, the size of the table explodes. This not only increases the memory requirements, but also makes exploration of the problem space infeasible. Deep RL algorithms address this issue by replacing the table with artificial neural networks (ANNs). These ANNs operate as function approximators, allowing the agent to make appropriate decisions with only partial exploration of the problem space. A variety of deep RL algorithms have been developed, and are still being developed; however, this study focusses on the application of RL to network management. This study utilises the deep Q-network (DQN) [6] algorithm, which is well known and common in related works, though the proposed approach can also use other deep RL algorithms.

## 5. Evaluation

### 5.1. Platforms

To assist in the development and evaluation of the proposed approach, this study utilised two software frameworks.

#### 5.1.1. LoRaWAN Simulator

Inspired by the work of Bor et al. [15], a Python based modelling framework has been designed using the SimPy [30] library. It facilitates the execution of many independent processes and is an appropriate platform for simulating and modelling a LoRaWAN. It uses a log distance path loss model, which incorporates shadow fading, as experienced in denser environments. Adjusting the path loss exponent and standard deviation of the normally distributed shadow fading component allows for the simulation of different transmission environments.

LoRa communication mechanisms are implemented following the Semtech LoRa Application Notes [31,32]. The simulation framework evaluates packet link margin, allowing packet collision to be tested. The base station model contains an eight channel radio, enabling reception on eight channels and transmission on one channel simultaneously.

#### 5.1.2. Reinforcement Learning Framework

To facilitate the RL component of this study, the Tensorforce [33] RL framework was used. Tensorforce is a Python library built on top of Tensorflow [34]. It supports some popular RL algorithms, and simplified the task of integrating them into applications. The modular structure of Tensorflow makes the substitution of the simulation framework with another, or even a physical network, which is possible without changing the RL components. Equally, the RL algorithm can be changed without affecting the simulation framework.

### 5.2. Experimental Set-Up

#### 5.2.1. LoRaWAN Configuration

This evaluation aims to represent a large scale urban network. This is a typical use case for LoRaWAN. Two application scenarios, smart metering and environmental monitoring, are the practical scenarios. LoRaWAN base stations typically have the ability of demodulating multiple channels simultaneously. From a network capacity perspective, each channel can be considered independently. A single channel simulation containing 128 nodes is equivalent to an 8 channel, 1024 node network, which is the actual scale used in this evaluation.

Both scenarios were simulated in a dense, urban environment, with high path loss and variability. This allowed the system’s responsiveness to network dynamics to be tested. In this case, network dynamics took the form of a co-located network introduced during the simulations. This was represented as noise, with a mean of 6 dB and log normal variance with a standard deviation of 6 dB. Table 1 lists the LoRa parameters which were common to both scenarios. The scenario-specific parameters are indicated in the following subsections.

#### 5.2.2. Agent Training

As is common for machine learning systems, the agent must be trained before online deployment. Node positions were randomly generated at the start of each episode, as were the scenario specific parameters: period, payload length, and application requirements. The RL training parameters are listed in Table 2.

### 5.3. Scenario 1—Environmental Monitoring

Scenario 1 represented a network of environmental monitoring sensors, a typical smart city application, where specific physical parameters such as temperature, humidity, and air quality, are monitored. The payload length was 8 Bytes, and the message period was 10 min. These nodes were battery powered, so this application prioritised ECP using the following initial application requirements: PDR=50%, ECP=80%.

**Figure 6:**
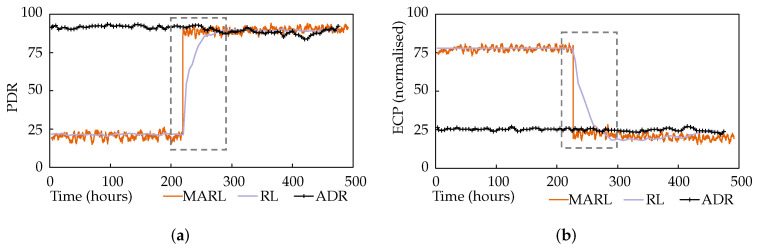
Scenario 1 results: (**a**) PDR performance for scenario 1. (**b**) ECP performance for scenario 1.

This experiment tested the application awareness feature of the proposed approach. During the simulation, a hypothetical alarm state occurred in the environment, prompting the nodes to change their application requirements to prioritise the timely delivery of packets. The application requirements became PDR=100%, ECP=5%. Figure 6a,b plot the average network PDR and ECP performance of the multi-agent reinforcement learning (MARL) approach against the performance of the single-agent approach [24], labelled as RL. A network utilising LoRa’s ADR mechanism is also shown.

A few observations can be made from these results. Firstly, while ADR always favoured PDR, regardless of any application requirements, the application awareness of the MARL and RL approaches is evident, with a clear preference for ECP performance during the first portion of the test, presented as an inverse normalised value in Figure 6b. Secondly, when the application requirements changed, both the MARL and RL changed their focus and promptly began experiencing performance in line with their new application requirements. Furthermore, finally, while the RL approach took 40 hours to complete the shift, the MARL approach achieved this in just 6 min, as highlighted by the dashed rectangles. This is because, in the single-agent system, the base station hosted agent would wait until it had a valid PDR measurement for every node before the iteration could be completed, and new parameters issued for the relevant nodes. In the multi-agent system, however, each agent can independently select and apply new parameters, cutting the iteration short, without waiting for the rest of the network. In comparison, ADR had static performance, favouring PDR without considering changing application requirements. This resulted in the node consuming more energy and having a shorter battery life.

ADR, while displaying adequate performance, has no application awareness.

**Figure 7:**
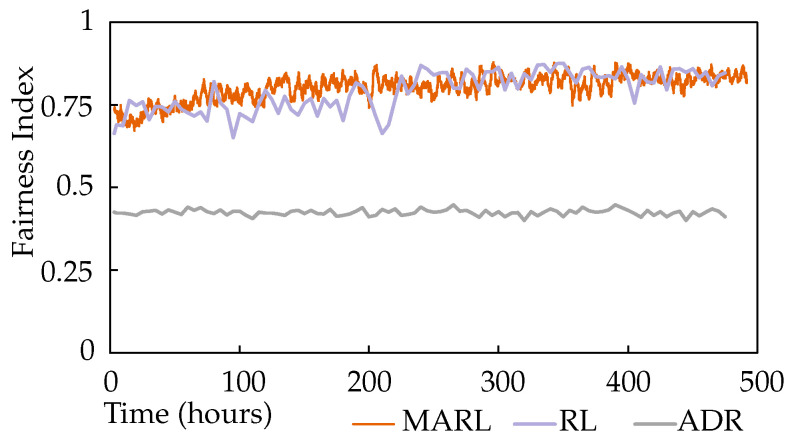
Scenario 1 fairness index for MARL.

Figure 7 shows the fairness index of the network over the same time scale. A gentle but steady increase in the fairness index can be observed for MARL, which is not impacted significantly by the change in application awareness. It is notable that the reward functions continued to encourage the improvement of network fairness when the individual node performance metrics settled. The same fairness index has also been plotted for the single-agent RL approach and ADR. ADR demonstrates significantly lower fairness, and the single-agent approach is less stable.

### 5.4. Scenario 2—Smart Meter

Scenario 2 illustrates the messaging between a network of smart power meters and a base station. The payload length was 12 Bytes, and the message period was 30 min. A smart power meter is unlikely to be battery powered, so this application gave priority to PDR over ECP. It used the following application requirements: PDR = 100%, ECP = 1%.

**Figure 8:**
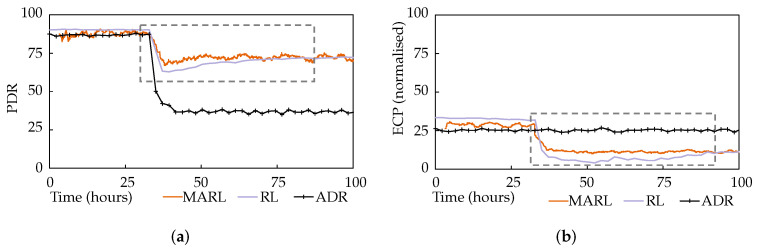
Scenario 2 results: (**a**) PDR performance for scenario 2. (**b**) ECP performance for scenario 2.

This experiment tested the system’s responsiveness to network dynamics. During the simulation, external interference was introduced representing the addition of a co-located network. This was simulated as significant noise with a mean of 6 dB and log normal variance with a standard deviation of 6 dB. Figure 8a,b plot the average network PDR and ECP performance, again alongside the single-agent and ADR approaches.

It is immediately obvious that ADR is ineffective at handling this interference. The MARL and RL approaches both performed similarly, with the MARL approach demonstrating a modest advantage in terms of responsiveness, highlighted by the dashed rectangles, though not as significantly as in scenario 1. The RL system’s PDR was initially 8% lower than MARL’s, though it caught up. Setting aside the responsiveness advantage of MARL, and after giving all approaches the time to settle post-interference, in can be seen that RL and MARL achieved 102% and 105% increases in PDR, respectively, compared to ADR.

**Figure 9:**
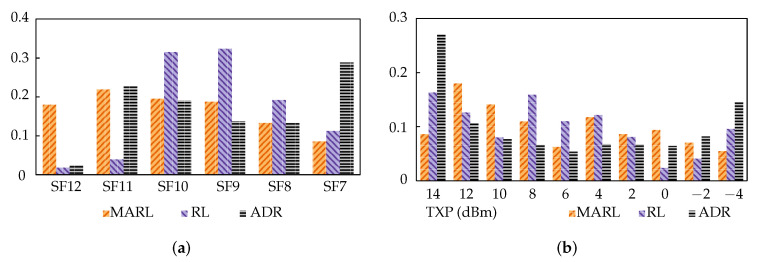
Scenario 2 results: (**a**) SF distribution for scenario 2. (**b**) TXP distribution for scenario 2.

Figure 9a,b illustrate the SF and TXP distributions of the three approaches post-interference. The y-axis indicates the proportion of nodes using the parameters specified on the x-axis (i.e., SF or TXP). ADR’s distribution is as expected of the algorithm, striving for just enough link margin, and SF reduction was favoured over TXP. This performed adequately in stable networks, but when there was significant, variable noise or congestion, it fell short. The most important aspect of this result is, however, the comparison between the MARL and RL approaches. Despite achieving similar performances, they had quite different parameter distributions, especially in SF. This shows that they follow different strategies, which is sensible given that they utilise different reward functions.

### 5.5. Discussion

From the results presented above, we can draw a few conclusions. The multi-agent reinforcement learning approach clearly demonstrates application awareness and the ability to tolerate network dynamics. In these regards, it clearly outperforms ADR. Compared with the single-agent approach, which also has the same goals, it demonstrates an advantage in terms of responsiveness.

More compelling than the quantitative results, however, are the qualitative benefits that the multi-agent approach offers over the single-agent. The distributed nature and significantly reduced search space make deployment of the agent on nodes possible. This naturally results in a node-centric approach. Nodes can control the trade-off between responsiveness and energy consumption, by modifying their learning iteration length. The selection of application requirements, or even reward functions, can be done on the node. Most significantly, however, is the independence of this approach from network size. Unlike a single-agent approach, the trained agent is not restricted to a fixed number of nodes, and the network can grow or shrink during operations, which is inevitable in real-world scenarios.

A limitation of the proposed approach, as it stands, is that it results in many independent nodes with similar experience which is not shared. If the state space was expanded to include a measure of node distance from the base station, perhaps with RSSI feedback from the base station to the node, the agent could be moderately generalised with respect to distance.

## 6. Conclusions

This study set out to address the issue of congestion in large scale wireless networks. Autonomy, adaptivity, application awareness, and fairness were identified as required features for a solution. A wireless network management framework has been proposed that incorporates multiple distributed deep RL agents, which utilise a novel reward function that satisfies node-specific application requirements and network fairness at the same time. Experimental results demonstrated autonomy, adaptivity, application awareness, and fairness, using two real-world scenarios. Additionally, the proposed approach benefits from network size independence, reduced search space, and node-led priorities, and supports variable iteration lengths.

Future work will include deployment on a physical network in order to validate the approach. To enable increasing scale, learning time will need to be reduced. Experience sharing is a potential direction for achieving this, as all nodes are then effectively working on the same problem independently, and their experiences are relevant to each other. The federated learning paradigm could be useful in this context when dealing with truly large networks. Experience sharing will require that agents be generalised with respect to distance from the base station, which necessitates expansion of the state space.

## Figures and Tables

**Table 1:** Common simulation parameters.

Parameter	Value
Nodes	128
Node Distribution	Random Uniform
Cell Radius	750 m
Spreading Factors	SF7 … SF12
Transmission Power Range	−4 dBm … 14 dBm
Transmission Power Steps	2 dB
Coding Rate	4/5
Frequency Band	915 MHz
Uplink Channel Bandwidth	125 kHz
Uplink Channel Spacing	200 kHz
Uplink Channels	1
Frame Length	Payload + 148 bits
Path Loss Exponent (λ)	3.5
Shadow Fading Std Dev (σ)	9

**Table 2:** RL training parameters.

Parameter	Value
Training length	500 episodes
Episode length	1000 iterations
Memory	100 episodes
Batch Size	4
Discount Factor γ	0.1
Exploration ϵ	0.1
Learning Rate	$e^{-3}$
Update Frequency	1

## Abbreviations

The following abbreviations are used in this manuscript:

ToA	Time on air
SF	Spreading factor
TXP	Transmission power
PER	Packet error rate
PDR	Packet delivery ratio
ECP	Energy consumption per packet
ADR	Adaptive data rate
RL	Reinforcement learning
DQN	Deep Q-network
LPWAN	Low power wide area network
IoT	Internet of Things
